# Image based prognosis in head and neck cancer using convolutional neural networks: a case study in reproducibility and optimization

**DOI:** 10.1038/s41598-023-45486-5

**Published:** 2023-10-24

**Authors:** Pedro Mateus, Leroy Volmer, Leonard Wee, Hugo J. W. L. Aerts, Frank Hoebers, Andre Dekker, Inigo Bermejo

**Affiliations:** 1 Department of Radiation Oncology (Maastro), GROW School for Oncology and Reproduction, Maastricht University Medical Centre+, Maastricht, The Netherlands; 2 Clinical Data Science, Maastricht University, Maastricht, The Netherlands; 3 Artificial Intelligence in Medicine (AIM) Program, Mass General Brigham, Harvard Medical School, Boston, MA USA; 4 Department of Radiology and Nuclear Medicine, Maastricht University Medical Center, Maastricht, The Netherlands; 5 Departments of Radiation Oncology and Radiology, Brigham and Women’s Hospital, Dana-Farber Cancer Institute, Harvard Medical School, Boston, MA USA

**Keywords:** Medical research, Biomarkers, Prognostic markers, Cancer, Cancer imaging, Computer science

## Abstract

In the past decade, there has been a sharp increase in publications describing applications of convolutional neural networks (CNNs) in medical image analysis. However, recent reviews have warned of the lack of reproducibility of most such studies, which has impeded closer examination of the models and, in turn, their implementation in healthcare. On the other hand, the performance of these models is highly dependent on decisions on architecture and image pre-processing. In this work, we assess the reproducibility of three studies that use CNNs for head and neck cancer outcome prediction by attempting to reproduce the published results. In addition, we propose a new network structure and assess the impact of image pre-processing and model selection criteria on performance. We used two publicly available datasets: one with 298 patients for training and validation and another with 137 patients from a different institute for testing. All three studies failed to report elements required to reproduce their results thoroughly, mainly the image pre-processing steps and the random seed. Our model either outperforms or achieves similar performance to the existing models with considerably fewer parameters. We also observed that the pre-processing efforts significantly impact the model’s performance and that some model selection criteria may lead to suboptimal models. Although there have been improvements in the reproducibility of deep learning models, our work suggests that wider implementation of reporting standards is required to avoid a reproducibility crisis.

## Introduction

The field of artificial intelligence, especially machine learning, has captured the interest of several sectors in recent years, including healthcare^1^. The substantial amount of data generated in this domain provided opportunities for models capable of assisting medical decisions, predicting outcomes, and moving in the direction of precision medicine^2^. Deep learning (DL), a machine learning technique, departed from traditional methods by promoting a complex structure capable of developing decision boundaries that outperformed previous approaches and, in some cases, specialists^3^. Within this field, a sub-class of deep neural networks, convolutional neural networks (CNN), has shown particular ability for processing imaging data by identifying predictive features without the need for feature engineering^4^.

In recent years, there has been a rapid increase in applications using CNNs in the medical field, taking advantage of the vast imaging data collected by healthcare centers. These applications span diverse goals, such as disease prediction and imaging segmentation^3^. Nevertheless, the complex structure that characterizes neural networks inhibits the explainability of their decisions^5^. In addition, CNNs generally require more data than traditional machine learning techniques and diverse sources^6^. Encouraged by its potential, this field has seen a growing number of published models. However, only a fraction gets applied in healthcare for reasons that include the lack of reproducibility and guidance to build robust imaging models.

The challenges in reproducing machine learning models unfolded as a prominent topic in the field. A large part of the studies does not provide enough information to achieve this goal^7^. This inability to reproduce a model directly impacts the study's replicability, preventing the model's generalization over different data. To overcome this problem, solutions, such as the TRIPOD^8^ statement, propose standard checklists to improve reporting transparency. Additionally to these guidelines, the technical aspects of the methods employed are crucial, as seen in a series of reports^7,9,10^ that identified the code and data unavailability as the primary factors contributing to this issue. Moreover, the necessary actions extend beyond these and include the inadequate specification of the pre-processing methods, model, training and evaluation procedures, software used, the selective report of results, and insufficient statistical details also play a critical role.

As one of the ten cancers with the highest incidence in the world^11^, head and neck cancer (HNC) presents heterogeneous aspects that hinder the attainment of successful treatment plans and precise prognosis^12^. Although there have been improvements in treatment and understanding of the disease, survival hasn't significantly improved in the last decades for the HNC population in general^12^, except for HPV-related cancers. Moreover, contributing to this concern is the occurrence of locoregional recurrence and distant metastasis, important outcomes that strongly affect the chances of survival^11,13^. Applying machine learning to assist the prognosis of these events has been the focus of several recent studies that presented promising results by extracting and learning from the information of the medical images available. One of these applications, published in 2017 by Diamant et al.^14^, consisted of a CNN developed for outcome prediction in head and neck cancer patients. The network used tumor delineations from the pre-treatment CT scans to predict distant metastasis, loco-regional failure, and survival. This work displayed the potential of DL tools by outperforming methods based on radiomics features, a traditional framework relying on feature engineering^15^.

Later, Lombardo et al.^16^ and Le et al.^17^ extended this study by exploring approaches to improve the model’s discriminative power and scope. Lombardo et al.^16^ included external validation datasets and performed a time-to-event analysis based on the CNN output. In their work, although demonstrating the predictive power for distant metastasis classification, the performance of the CNN was notably lower. Furthermore, the inclusion of clinical variables improved the network's performance. Le et al.^17^ evaluated adding modules to handle the data variability between institutions, using all slices available with tumor tissue and incorporating PET information. The results, similar to Lombardo et al.^16^, showed a lower performance for distant metastasis prediction using an identical network to the one described by Diamant et al.^14^ However, their proposed network displayed better results for loco-regional failure and overall survival prediction. Additionally, the findings of this work suggested that the CNN benefits more from the inclusion of clinical information than the PET scan. Nonetheless, the performance remained lower than in the study proposing the CNN, a problem later faced by the authors when trying to reproduce their work.

This work focuses on the factors that commonly impact the reproducibility of studies proposing CNN applications. We accomplished this by assessing previous studies for HNC outcome prediction based on aspects identified in the literature that pose challenges to reproducible work. It is our hypothesis that these aspects are indeed crucial for reproducible science. Furthermore, we developed a CNN framework centered on these factors, compared the results with the previous studies, and evaluated the impact of imaging pre-processing approaches for medical images and model selection on a CNN performance.

## Methods

### Reproducibility assessment

Evaluating the reproducibility of a study takes into account several aspects that impact the ability to implement its model and achieve the results reported. In this study, different aspects reported in studies^7,9,10^ on this topic combined with checklists^18,19^ for clinical artificial intelligence models were used to assess the reproducibility of previous studies in HNC prognosis using DL. Furthermore, these aspects supplied a guideline to the DL model presented in this study. The resulting checklist extends over three domains proposed by McDermott et al.^7^, technical reproducibility, statistical reproducibility, and generalizability. The first mainly encompasses the detailed description of the model, the datasets, and the release of the code. Statistical reproducibility evaluates the quantification of the model performance with measures of central tendency and uncertainty. Lastly, generalizability accounts for evaluating the model with an external dataset uninvolved in the training and validation process. Extending the model evaluation to unbiased data gives a better understanding of the model's generalizability and the potential to replicate it. In addition, we completed this assessment by employing the Checklist for Artificial Intelligence in Medical Imaging (CLAIM)^19^ for each study.

In this assessment, we looked into the work of Diamant et al.^14^, Lombardo et al.^16^ and Le et al.^17^, three studies proposing a CNN models evaluated with publicly available data. For this, we followed the specifications given by the authors, retrieved the data from its sources, and attempted to train the model with the publicly available code. Besides, we contacted the respective authors for additional information. Diamant et al.^14^ and Le et al.^17^ trained and evaluated a model separately for each outcome: distant metastasis, loco-regional failure, and overall survival. On the other hand, Lombardo et al.^16^ focused exclusively on exploring one model for distant metastasis occurrence prediction. In our work, we assessed these models' reproducibility within the proposed objectives from their original work.

### Data

The data used in this study consisted of de-identified pre-treatment CT scans obtained from 435 patients diagnosed with head and neck cancer without metastasis at the time of diagnosis from two distinct publicly available datasets at the The Cancer Imaging Archive (TCIA)^20–22^. The first cohort^21^, obtained from four different institutions in Canada, accounted for 298 patients after excluding cases with errors in the initial data curation. The second cohort^22^, obtained from one institution in the Netherlands, contained 137 patients. In both cases, an expert performed the 3D gross tumor volume (GTV) delineations as part of the routine clinical workflow in radiation treatment and the data available consisted of DICOM images and radiotherapy structures. Both datasets included a set of variables alongside the images including the patient's age, biological sex, HPV status, tumor location, T, N, M, and overall staging (according to the 7th Edition of the cancer staging manual by the American Joint Committee on Cancer^23^), treatment, and outcomes. In addition, we extracted the GTV area and volume from the imaging metadata.

The patients' data available in each cohort included the time in days to each outcome (event time) and the follow-up time. In this study, to handle the limitations posed by using right-censored data, the patients with a follow-up time below the defined event time frame were excluded (overview of the number of patients shown in Supplementary Table 2). Specifically, the time frame considered in this study for distant metastasis and loco-regional failure prediction was the commonly used 2 years since most occurrences happen during this period^24,25^. In the case of overall survival, a 4-year time frame, the follow-up time median, was used instead of the conventional 5-year interval^11,24^ to avoid excluding 47 additional patients.

The data split for training and validation was performed using two different methods. For reproducibility purposes, one approach followed the same distribution as the one implemented by Diamant et al.^14^ (cohort split): the data from two Canadian institutions in each, comprising 192 and 106 patients, respectively. The second method consisted of performing 5-fold cross-validation (CV) using all Canadian institutions for training and validation. Additionally, the dataset from the Dutch cohort was used exclusively as the testing set to evaluate model generalization. Table 1 provides a complete overview of both data split methods (complementary patient characteristics shown in Supplementary Table 1).

**Table 1:** Patient and outcome distribution across the different centers.

		Cohort split			5-fold CV	
		Training	Validation	Testing	Training and validation	Testing
Data sources						
Canadian institutions^21^	HGJ	91 (47.6%)	–	–	91 (30.6%)	–
	CHUS	100 (52.4%)	–	–	100 (33.7%)	–
	HMR	–	41 (38.7%)	–	41 (13.8%)	–
	CHUM	–	65 (61.3%)	–	65 (21.9%)	–
Dutch institution^22^	Maastro	–		137 (100%)	–	137 (100%)
Outcome						
	Distant metastasis (DM)	26 (13.6%)	14 (13.2%)	8 (5.8%)	40 (13.5%)	8 (5.8%)
	Loco-regional failure (LRF)	27 (14.1%)	16 (15.1%)	34 (24.8%)	43 (14.5%)	34 (24.8%)
	Death	32 (16.8%)	24 (22.6%)	74 (54.0%)	56 (18.9%)	74 (54.0%)
	Total	191	106	137	297	137

*CHUM* Centre Hospitalier de l’Université de Montréal, *CHUS* Centre Hospitalier Universitaire de Sherbooke, *HGJ* Hôpital Général Juif, *HMR* Hôpital Maisonneuve-Rosemont.

### Pre-processing

In both cohorts, the primary GTV delineations, performed by an experienced oncologist in the CT scans and provided as DICOM RTSTRUCT, and the DICOM images were resampled to a uniform pixel spacing (1 × 1 mm^3^), calibrated to Hounsfield units (HU), and transformed to the NIFTI format using the “dcmrtstruct2nii”^26^ python library. Moreover, FSL^27^, a library of analysis tools for brain imaging data, allowed to re-orient the scans to the MNI152^28^ standard template and apply the GTV masks to obtain the scans’ portion containing the region of interest. For each participant, a single CT slice was selected by identifying the one with the largest GTV area from the resulting stack of CT slices.

The resulting NIFTI images were posteriorly transformed by windowing the pixel values according to the Hounsfield scale, smoothed with a Gaussian filter, and normalized to a scale from 0 to 1. To explore the impact of windowing CT images, we considered different windowing parameters and compared them based on the model’s performance. As a starting point and based on the previous studies, images were windowed using a level of 0 HU and a width of 1000 HU. Additionally, we explored using a window level of 50 HU and a width of 350 HU based on the expected interval of the Hounsfield scale for the tissues in the head and neck region (e.g., mucosal, soft tissues)^29^.

Analyzing the GTV area led to cropping the images around the tumor center from the standard CT size of 512 × 512 pixels to a smaller region, enhancing the learning process without losing information. Based on inspection of the GTV sizes, the dimensions used consisted of 180 × 180 pixels. Finally, the images were stored in an 8-bit Portable Network Graphic (PNG) format limiting the range of values to 255 integers.

### Model description

**Figure 1:**
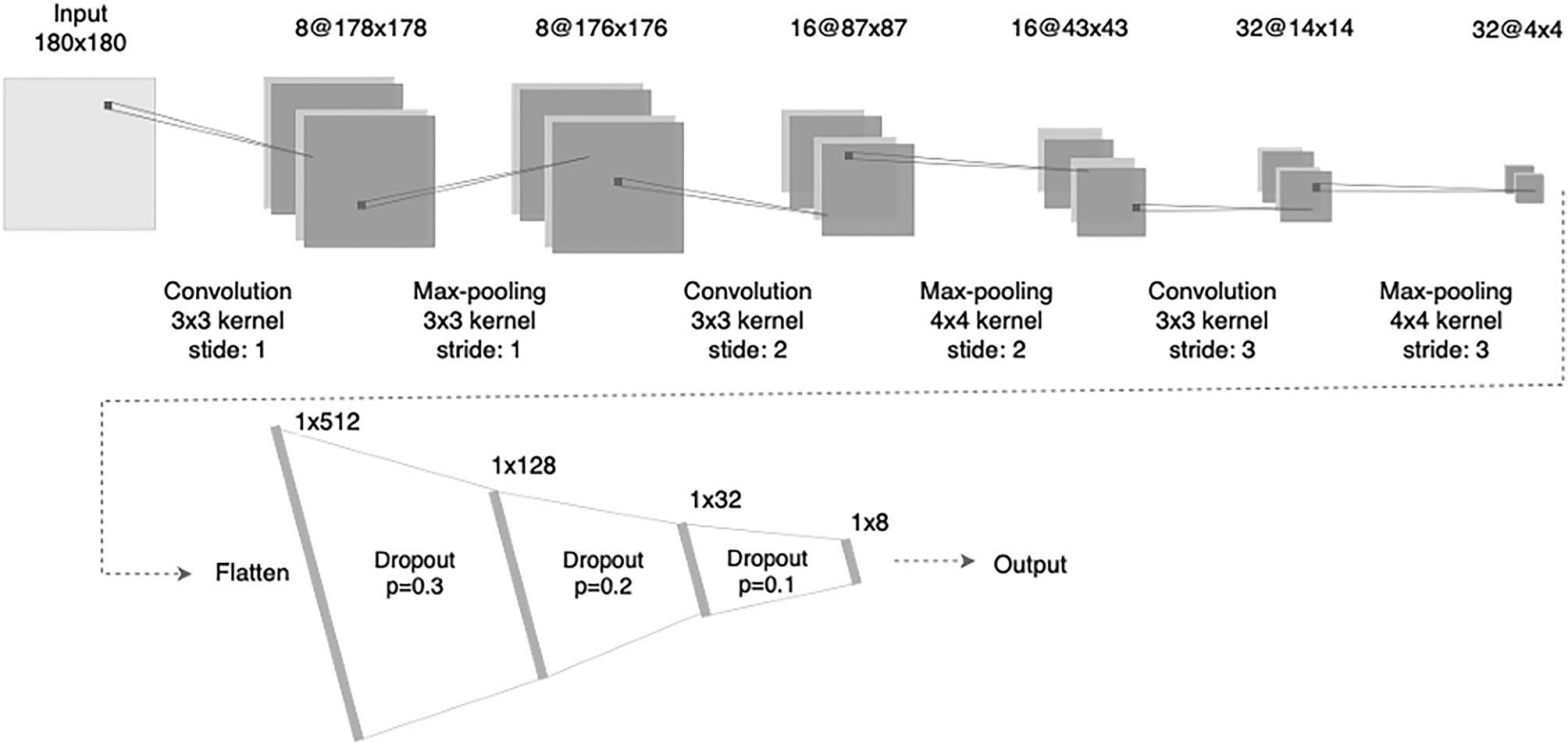
CNN architecture adapted from Diamant et al.^14^ for outcome prediction. The description includes the number of neurons for the fully connected layers and the number of filters, kernel size, and stride for each convolutional block.

The basis for the CNN architecture consisted of the structure proposed by Diamant et al.^14^, a network that accepted as input a standard CT image, 512 × 512 pixels. However, in light of the inability to reproduce their results, we propose an adaptation to the network structure, represented by Fig. 1, using as input a cropped region of 180 × 180 pixels from the original scan around the tumor center. This approach, suggested but not applied by Diamant et al.^14^, can be easily integrated into the pre-processing, as seen in the work of Lombardo et al.^16^. It facilitates the learning process by reducing the model's number of parameters considerably. Furthermore, we assessed simplifying the network structure by evaluating a smaller number of filters, fully connected layers, and the application of dropout to reduce overfitting. The resulting network consists of two segments, the first with three convolution blocks, each applying a convolution layer, a max-pooling layer, and a non-linear transformation using the leaky rectified linear unit (leaky ReLU) function. These operations transform the pre-processed scan into a 4 × 4 image embedding with 32 channels. Taking these 512 features as input, the second segment consists of four fully connected layers, each using a linear transformation, a non-linear transformation, the leaky ReLU function, and a dropout layer. The last component consists of a sigmoid function to transform the result into a binary prediction.

To evaluate the impact of the structured data available (i.e., age, gender, TNM stage, etc.) on the model's performance, we developed a second model that includes these variables as input. This additional data provides relevant information on the patient and tumor characteristics that clinicians use for prognosis and treatment choices^11,25^. Non-imaging data can be included in a CNN using the fully connected layers, and, as a result, multiple locations are possible. In this study, we selected the clinical variables to add to the model using forward feature selection, adding in each step the variable that provided the highest performance increase in the validation set, and assessed the integration in the network by evaluating the model's performance when including these variables at each of the fully connected layers. Regarding the clinical data, we categorized the volume and area according to the quartiles, used one-hot encoding to encode the categorical variables, and normalized the age considering a maximum of 100. Additionally, we aggregated the cancer staging variables according to the main categories (e.g., T4 represented T4a and T4b). The M staging information was not included since the criteria for the data sources excluded patients with metastases at presentation.

### Artificial neural network

In order to assess the predictive power of the structured data and measure the added value of imaging features, we trained an artificial neural network (ANN) using only the structured data. This ANN consisted of four layers with an input layer with 11 neurons and two hidden layers with eight and four neurons respectively and the output layer. We used the same hyperparameters and components as in the previous model, using the leaky ReLU as activation function as and a sigmoid function for the output binary prediction. Furthermore, a logistic regression model was used as baseline to evaluate the performance of both the CNN and ANN models for each outcome prediction.

### Model evaluation and selection

The model evaluation and selection relied primarily on the ROC (Receiver Operating Characteristic) AUC (Area Under the Curve) metric. This measurement, agnostic to a threshold selection, evaluates a binary model’s discriminative power, i.e., its ability to distinguish between two classes.

For both data partition approaches, uncertainty measurement, using the confidence interval for the cohort’s split and range of values for the 5-fold CV, complemented the results. The 95% confidence interval was calculated for the selected model using a bootstrapping technique with resampling of the data in each set (1000 resamples). Furthermore, the cross-validation employed a stratified sampling technique taking into account the imbalanced nature of the data and maintaining the outcome proportions constant in each fold.

We selected the model at the epoch with the highest ROC AUC in the validation set from the epochs where the difference between the training and validation ROC AUCs was below a certain threshold to avoid overfitting. For this study, the initial threshold used was 0.05, gradually increasing the value until a model meeting the requirement was found.

### Experimental setup

In terms of software tools, we used FSL, Docker, and Python 3.9. For the pre-processing, FSL allowed to extract the region of interest from the CT scans, followed by the cropping and windowing operations performed using a Python script. Regarding the neural networks, development and optimization was performed using PyTorch (version 1.10.0)^30^. Additional libraries employed for image augmentation and performance evaluation are described in the public repository with the respective versions.

Considering the limited size of the dataset, data augmentation was used to avoid overfitting. The images were randomly flipped on the horizontal and vertical axis, with a probability of 0.5, rotated 90° a random number of times, additionally rotated by a value within the range of 0°–20°, and shifted on the horizontal and vertical axis 3% of the total width and length respectively. Moreover, we manually tweaked the network and training hyperparameters based on the output of “Weights and Biases”^31^. Furthermore, we included a weighting term in the loss function to adjust for class imbalance and initialized the model's weights with the default PyTorch method, employing the He initialization^32^. Lastly, the network was optimized using stochastic gradient descent with a batch size of 64 samples.

The network’s training process was executed on a CPU cluster hosted on a Kubernetes infrastructure, a distributed computing platform based on containers, with 64 cores and 512 GB of memory available. The network was trained for 3000 epochs with early stopping when it reached an AUC superior to 0.95 for the training data. In each experiment, a model was trained for each outcome separately.

In addition to the code used for this study, the public repository includes the necessary tools to recreate an identical environment. By using Docker, a containerization mechanism, it is possible to perform the training and evaluation of the CNN following the software specifications described in this section to reproduce the results described.

## Results

### Reproducibility assessment

The reproducibility assessment, presented in Table 2, displays the compliance of the previous studies^14,16^ on head and neck cancer prognosis using DL and our work with the criteria previously described and the CLAIM^19^ checklist (evaluation provided in the supplementary materials). In this assessment, Diamant et al.^14^and Le et al.^17^ missed elements that preclude reproduction of the results, such as the specification of data pre-processing or a complete and functional code release. Moreover, the three studies missed at least one element, mainly the environment description or disclosure of the random initializers, that prevented the reproduction of the exact results presented. Concerning the statistical reproducibility, all studies provide most of the necessary information, except for the evaluation of uncertainty in the work of Diamant et al.^14^. The uncertainty around the performance estimates can be represented, for example, as a confidence interval and is essential information to compare performances across studies. Lastly, conceptual reproducibility, comprising the external validation of the model, was assessed by Lombardo et al.^16^ using three external datasets and by Le et al.^17^ employing a cross-validation strategy with the Canadian institutions.

**Table 2:** Results of the reproducibility assessment.

	Diamant et al*.*^14^	Lombardo et al*.*^16^	Le et al.^17^	Our work
Technical reproducibility				
Network architecture	✓	✓	✓	✓
Hyperparameters evaluation and selection	✓	✓	✓	✓
Model evaluation	✓	✓	✓	✓
Model selection		✓	✓	✓
Pre-processing specification		✓^a^		✓
Censored data handling			✓	✓
Data split specification	✓	✓	✓	✓
Code release	✓^b^	✓	✓	✓
Environment description (libraries versions)		✓		✓
Computational infrastructure description	✓	✓	✓	✓
Dataset(s) publicly available	✓	✓	✓	✓
Reproducible pipeline^c^				✓
Random seed				✓
Statistical reproducibility				
Evaluation of central tendency	✓	✓	✓	✓
Evaluation of uncertainty		✓	✓	✓
Cross validation/bootstrapping	✓	✓	✓	✓
Generalizability				
Performance metrics on all data partitions				✓
External validation		✓	✓	✓
CLAIM^19^ completeness	74%	88%	86%	95%

^a^Code provided upon request to the authors.

^b^Code publicly available but not fully functional.

^c^As specified in “Part 6: reproducible pipeline” of the MI-CLAIM checklist by Norgeot et al.^18^.

Altogether, we could not reproduce Diamant et al.’s work. We had difficulties figuring out the correct library versions, the image pre-processing was not reported, and we had issues with the convergence of the model, leading to an estimated performance significantly lower than the one detailed in their article (AUC of 0.79 versus the reported 0.88). On the other hand, Lombardo et al.^16^ provided the necessary tools to train the model, resulting in an identical performance for the CNN based on imagining data. Furthermore, we observed that in both studies the data augmentation methods cropped the images within the GTV region. Lastly, we could not reproduce the work proposed by Le et al.^17^ because of the incomplete documentation regarding the input data preparation and the absence of details for the image pre-processing.

During this work, we contacted the corresponding authors of each study for additional information. We did not obtain a response from Diamant et al.^14^, Lombardo et al.^16^ provided the code for the CT scans pre-processing according to their shape-based interpolation method, and Le et al.^17^ informed us that the code is currently being prepared for release.

### Model architecture and training

The model’s optimization encompassed a range of values for the model’s hyperparameters with similar performances. The best-performing model resulted when employing a constant learning rate of 0.05, an L2-regularization parameter of 1 × 10^−4^, a momentum of 0.9, and a slope coefficient of 0.01 for the leaky ReLU. The weighting terms were identical for the three events predicted, with a rescaling factor of 3.7 for the minority class and 0.7 for the dominating class. The CNN proposed included 85,505 trainable parameters, 96.3% less than in Diamant et al.’s work (2,316,385 parameters) and 87.8% less than in Lombardo et al.’s work (692,298 parameters). The network’s training time was approximately 3 h, requiring a maximum memory usage of 3 GB.

### Comparative performance

The performance of our network varied for different outcomes, as shown in Table 3: the 2-year distant metastasis prediction had the highest AUC, around 0.90, across the training, validation, and testing sets. These results are similar to those reported by Diamant et al.^14^ and superior to those reported by Lombardo et al.^16^, especially in the validation set, regardless of the type of validation. In terms of 4-year overall survival and 2-year loco-regional failure prediction, our model performed better than Diamant et al.’s original work in the validation set: our CNN achieved an AUC 0.78 and 0.77, for overall survival and for loco-regional failure prediction, respectively, compared to the AUCs of 0.65 and 0.70 reported by Diamant et al.^14^*.* However, in both cases, the AUCs achieved by our model plummeted in the test set, to 0.67 for overall survival and to 0.45 for loco-regional failure, resulting in a complete loss of discriminative power for loco-regional failure prediction. Diamant et al.^14^ did not report the performance in a test set. Nevertheless, we observed a similar outcome in our attempt to reproduce their study using the same test set. Noticeably, the training set underperformed with AUCs closer to a random prediction.

- CNN with clinical data and ANN

**Table 3:** Comparative performance (AUCs) for different outcomes of the reproduced studies and our proposed CNN.

	Diamant et al*.*^14^		Lombardo et al*.*^16^	Le et al*.*^17^	Our CNN	
	Cohort Split	5-fold CV	3-fold CV	Cohort split (CI 95%)^c^	Cohort split (CI 95%)	5-fold CV
		Mean (range)	Mean (range)			Mean (range)
Distant Metastasis (2 years)^d^						
Training	–/0.70^a^	–	–/0.71^a^	–	0.91 [0.84, 0.96]	0.87 (0.84–0.92)
Validation	0.88/0.79^a^	0.85 (0.80–0.88)	0.75 (0.67–0.83)/0.73^a^	0.84 [0.83, 0.85]	0.89 [0.81, 0.96]	0.86 (0.77–0.96)
Testing	–/0.75^a^	–	0.81 [0.73–0.89]^b^/0.75^a^	–	0.89 [0.79, 0.98]	0.83 (0.76–0.90)
Loco-regional failure (2 years)^d^						
Training	–/0.52^a^	–	–	–	0.76 [0.64, 0.88]	0.77 (0.72–0.86)
Validation	0.65/0.61^a^	–	–	0.72 [0.67, 0.76]	0.77 [0.58, 0.92]	0.76 (0.72–0.84)
Testing	–/0.44^a^	–	–	–	0.45 [0.32, 0.57]	0.53 (0.48–0.59)
Overall survival (4 years)^d^						
Training	–/0.55^a^	–	–	–	0.84 [0.75, 0.92]	0.82 (0.68–0.94)
Validation	0.70/0.67^a^	–	–	0.77 [0.75, 0.79]	0.80 [0.66, 0.91]	0.77 (0.62–0.96)
Testing	–/0.58^a^	–	–	–	0.67 [0.57, 0.77]	0.63 (0.57–0.72)

^a^Reproduced for this study.

^b^Median (CI 83%).

^c^CI calculated over 5 trials.

^d^Event time may be different in the studies included.

Integrating the clinical data into the CNN did not consistently lead to significant improvements in the models’ AUC as shown in Table 4. The performance on the testing set for distant metastasis (AUCs 0.89–0.93) and overall survival (AUCs 0.67–0.69) prediction remained similar. However, it did result in improvements on the testing set for loco-regional failure (AUCs 0.45–0.59). We achieved the best results (which were comparable to our model based only on imaging data) when adding the clinical data in the network's last connected layer. The clinical variables that maintained the performance were mainly the T and N stages, and the volume discretized according to the quartiles.

The CNN outperformed the ANN in the test set on loco-regional failure (AUCs 0.59 vs 0.41), overall survival (AUCs 0.69 vs 0.63) and distant metastasis (AUCs of 0.93 vs 0.87) prediction. The logistic regression model achieved similar AUCs for the test set to the CNN in overall survival but lower in distant metastasis and loco-regional failure (results shown in Supplementary Table 4). Moreover, both the ANN and logistic regression underperformed in the training and validation sets compared to CNN (Table 4).

- Image pre-processing

**Table 4:** AUCs of models including clinical data.

	Lombardo et al*.*^16^	Le et al*.*^17^ ^c^	Our CNN		Our ANN	
	3-fold CV	Cohort split (CI 95%)^d^	Cohort split (CI 95%)	5-fold CV	Cohort split (CI 95%)	5-fold CV
	Mean (range)			Mean (range)		Mean (range)
Distant metastasis (2 years)^e^						
Training	–/0.84^a^	–	0.91 [0.86, 0.95]	0.88 (0.81–0.93)	0.87 [0.78, 0.93]	0.87 (0.81–0.92)
Validation	0.81 (0.73–0.86)/0.79^a^	0.80 [0.77, 0.83]	0.89 [0.79, 0.98]	0.87 (0.79–0.94)	0.79 [0.65, 0.93]	0.83 (0.79–0.88)
Testing	0.86 (0.79–0.92)^b^/0.86^a^	0.69 [0.68, 0.70]	0.93 [0.86, 0.99]	0.88 (0.86–0.90)	0.87 [0.78, 0.95]	0.86 (0.81–0.89)
Loco-regional failure (2 years)^e^						
Training	–	–	0.84 [0.76, 0.93]	0.77 (0.62–0.87)	0.71 [0.61, 0.80]	0.74 (0.70–0.84)
Validation	–	0.79 [0.77, 0.80]	0.70 [0.54, 0.84]	0.72 (0.60–0.84)	0.66 [0.48, 0.82]	0.71 (0.60, 0.81)
Testing	–	0.69 [0.68, 0.70]	0.59 [0.47, 0.70]	0.57 (0.53–0.60)	0.41 [0.29, 0.54]	0.53 (0.50, 0.54)
Overall survival (4 years)^e^						
Training	–	–	0.74 [0.64, 0.84]	0.83 (0.74–0.94)	0.83 [0.74, 0.90]	0.83 (0.77–0.85)
Validation	–	0.82 [0.80, 0.84]	0.74 [0.58, 0.86]	0.81 (0.73–0.93)	0.75 [0.62, 0.87]	0.76 (0.71–0.78)
Testing	–	0.69 [0.68, 0.70]	0.69 [0.59, 0.79]	0.68 (0.63–0.71)	0.63 [0.52, 0.73]	0.63 (0.61, 0.64)

^a^Reproduced for this study.

^b^Median (CI 83%).

^c^Testing on a different dataset from the CHUM.

^d^CI calculated over 5 trials.

^e^Event time may be different in the studies included.

**Figure 2:**
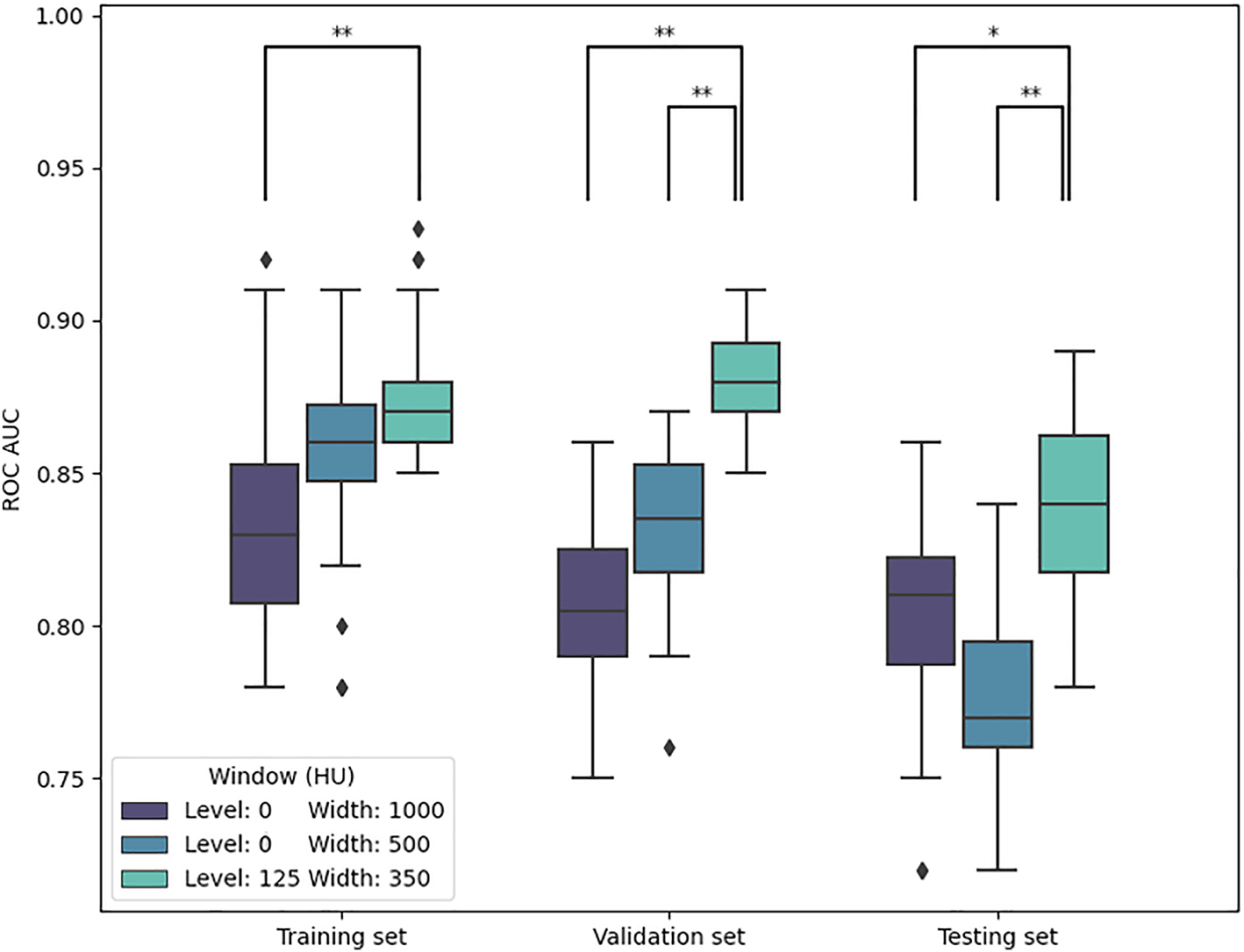
Model performance for 2-year distant metastasis prediction with different windowing and pre-processing options (**p < 0.001, *p < 0.05).

Figure 2 shows the model performance obtained from repeating the model training for 2-year distant metastasis prediction with varying parameters for the windowing step of the pre-processing pipeline. The model performed the best when using a window level of 125 HU and a width of 350 HU to preprocess the images. This model achieved an average AUCs of 0.88, 0.87, and 0.85 in the training, validation, and testing sets, respectively. For the wider window, using a window level of 0 HU and a width of 1000 HU, the AUCs were lower (0.83, 0.81 and 0.80). Similarly, applying a narrower window, with a width of 500 HU and a level of 0 HU, resulted in AUCs of 0.86, 0.83 in and 0.78 in the training validation set, and testing set, respectively. Statistically significant differences (Kruskal–Wallis, *p* < 0.05) were found for the validation and testing sets when comparing the AUCs of the best-performing window to the AUCs of models trained using the other two windows.

- Model selection criteria

Table 5 displays the AUC achieved in the training, validation, and testing sets for each predicted outcome and different model selection criteria. The model’s performance in the test set, in terms of AUC, differed under different model selection criteria for all outcomes. For distant metastasis, our selection criteria (described in the “Methods” section) achieved similar results to the model with the highest validation AUC and they both outperformed the model with the lowest training loss. For loco-regional failure and overall survival, all three model selection criteria resulted in similar results, but our selection criteria slightly outperformed the other two. Selecting the model based on the highest AUC for the validation set resulted in higher differences between the training and validation AUCs.

**Table 5:** Model performance for different model selection criteria.

	Our selection criteria	Highest validation AUC	Lowest validation loss
Distant metastasis			
Training	0.90 [0.82, 0.96]	0.82 [0.70, 0.91]	0.91 [0.83, 0.97]
Validation	0.90 [0.79, 0.97]	0.92 [0.84, 0.99]	0.90 [0.82, 0.96]
Testing	0.86 [0.72, 0.97]	0.86 [0.70, 0.98]	0.75 [0.57, 0.90]
Loco-regional failure			
Training	0.75 [0.66, 0.88]	0.53 [0.35, 0.68]	0.76 [0.64, 0.88]
Validation	0.71 [0.52, 0.88]	0.83 [0.67, 0.94]	0.64 [0.42, 0.82]
Testing	0.57 [0.45, 0.70]	0.54 [0.43, 0.66]	0.54 [0.40, 0.68]
Overall survival			
Training	0.76 [0.64, 0.87]	0.54 [0.41, 0.68]	0.78 [0.66, 0.88]
Validation	0.72 [0.57, 0.84]	0.77 [0.63, 0.9]	0.66 [0.49, 0.81]
Testing	0.72 [0.62, 0.80]	0.68 [0.58, 0.78]	0.70 [0.61, 0.79]

The values reported represent the ROC AUC with the 95% confidence interval in brackets.

## Discussion

In this study, we have tried to reproduce the results of three published models predicting outcomes for patients with head and neck cancer, as well as trying to optimise the performance of the model by testing different pre-processing options and model selection criteria.

Similar to previous studies^16,17^, we were unable to reproduce Diamant et al.’s work and results. On the other hand, we were more successful reproducing the results reported by Lombardo et al.^16^ but encountered difficulties that impeded reproducing Le et al.^17^ work. It can be challenging to guarantee the reproducibility of a DL model due to the experimental and complex nature of developing the network. In this study, we found difficulties across different domains when attempting to reproduce a DL model: environment configuration, pre-processing steps, random seed, weight initialization, data augmentation, statistical comparison, etc. Additional aspects, such as insufficient information regarding patient inclusion and event times, posed a barrier to guaranteeing a similar patient distribution. In agreement with previous studies^7,9,10^, we consider a set of reporting requirements necessary to guarantee such reproducibility, as shown by the inability to reproduce one of the studies. The prospect of developing a fully reproducible work can be improved by following one of the existing reporting checklists^8,18^ that aggregate these requirements. In this study, we went beyond the checklists by providing a complete specification of the random initializers employed and a reproducible pipeline. As suggested by Norgeot et al.^18^, we developed this pipeline using Docker, configured with the exact environment requirements necessary, the scripts and configurations implementing the model, and a subsample of examples that can facilitate an accurate replication of the proposed methods. Although there is a stochastic component to a neural network optimization process, in most cases existing technologies enable the reproduction of the exact results presented in a study^30^. Overall, the difficulties encountered revealed the need for auxiliary material, in addition to the scientific manuscript, to thoroughly describe the methodologies applied, reinforcing the importance of sharing the complete code.

While attempting to reproduce Diamant et al.’s results, we tried different techniques to maximize the model performance, such as optimizing the hyperparameters and image pre-processing steps. Eventually, this process led us to improve upon the CNN structure proposed by Diamant et al.^14^, decreasing the network's complexity while achieving similar results for distant metastasis and improving the results for loco-regional failure and overall survival in the training and validation datasets. Similarly, our model outperforms Lombardo et al.’s and Le et al.’s ^16,17^ predicting distant metastasis. However, the performance of our model plummeted in the external dataset for loco-regional failure and overall survival prediction. Le et al.^17^ reported similar drops in performance in their external validation and we observed the same phenomenon using Diamant et al.’s model (Lombardo et al.^16^ did not consider these outcomes). We believe this issue could be attenuated to some extent by increasing the sample size of the training data and including data from a wide range of clinics. Nevertheless, these findings may imply pertinent issues, such as a concept shift^33^ between the institutions or differences in the CT acquisition parameters, which may be relevant to explore in future research. In any case, these findings accentuate the importance of external validation for thoroughly evaluating a model as well as the need for further research to make these models less prone to overfitting and more generalizable.

Including clinical features in the CNN did not always enhance the model's performance. However, our preliminary results indicate a possible increase in the clinical features' relevance when considering longer time windows for the events. In addition, including clinical features did lead to better results in the test set for loco-regional failure. These results suggest that clinical features provide higher generalizability to the model for certain outcomes. Previous findings from Lombardo et al.^16^ and Le et al.^17^ differed on the impact of adding clinical features to their CNN models. However, studies showed that these attributes present interactions^11^ and correlate with the incidence of the outcomes explored^13,34^, possibly requiring more patients to evaluate the contribution of these features thoroughly. On the other hand, a neural network relying exclusively on the clinical data displayed a lower performance for distant metastasis and loco-regional failure prediction, showing the value of the imaging features identified by the CNN to improve the decision boundary. These findings provide insights into the potential of extending CNNs with clinical data, which may only be beneficial for specific outcomes.

The modifications introduced to the network structure proposed by Diamant et al.^14^, in particular the reduced image size and number of filters in the network, decreased the complexity of the network and demanded fewer resources without compromising the performance. Additionally, the data augmentation methods employed during training avoided cutting the tumor region, and a weighting term was included to counteract the class imbalance. In our work, the strategy to handle right-censored data consisted of excluding the cases without the minimum follow-up time. However, it is possible to extend the network with survival analysis to include censored data, as Lombardo et al.’s work showed by feeding the CNN’s output to a survival model. Another aspect to consider in future work is interpretability, explored by Diamant et al.^14^ through the association between radiomic features and the convolutional layers, which can be further studied through heatmap visualization or adaptations of the CNN^35^.

An important limitation of our model is that it processes one single slice of the pre-treatment CT (the one with the largest tumor area), ignoring several slices where the tumor is visible that may contain relevant information. Results from recent studies on outcome prediction^16,36^ and classification^37–39^ using 3D and 2D CNNs for head and neck patients demonstrated improved results when using a 3D inputs or both. However, the trade-off between the improvements and computational resources is still unexplored. Our network can be further extended to process a 3D image and understand these compromises.

The flexibility of CNNs can attenuate the differences in the pre-processing pipelines employed^40^. However, preprocessing choices can still significantly impact the model's performance, as shown by the results obtained with different windowing parameters applied to the CT scans. In contrast to humans, machine learning models can process the complete range of values with no transformations. Nevertheless, our results showed that determining the windowing parameters according to the target tissue can enhance the CNN’s ability of discerning relevant imaging features.

In DL, the available data is typically split into training, validation (or development) and testing datasets: the model’s parameters (i.e., weights) are determined by the training set, its hyperparameters by the validation set (i.e., model selection) and its performance is estimated on the test set. The training set is typically the largest, and the model’s performance on this set is essential to understand if the model may be overfitting or underfitting the data. In our work, we introduced a model selection criterion based both on the validation and training sets' performance. We observed that models selected based only on the validation set were sometimes underfitted to the training set, which had implications on the performance in the testing set. These results highlight the importance of reporting the metrics for all subsets of data and considering the training set performance for the model selection.

In conclusion, the results of our study show the importance of complying with reporting guidelines for the reproducibility of DL studies. They also show that model architecture, image processing decisions, additional clinical data and model selection criteria can have a significant impact in the model’s performance. Our work followed the guidelines for a reproducible network and achieved results that equaled or surpassed previous studies keeping a simpler structure. This work supports the potential of CNNs to extract imaging features with clinical relevance for head and neck cancer outcome prediction but also hints at necessary improvements for their generalizability.

### Supplementary Information

Supplementary Information 1.

Supplementary Information 2.

## Data Availability

The datasets analyzed during the current study are publicly available at the Cancer Imaging Archive (TCIA)^20^ repository: Canadian benchmark dataset^21^: https://doi.org/10.7937/K9/TCIA.2017.8oje5q00. MAASTRO dataset^22^: https://doi.org/10.7937/tcia.2019.8kap372n.

## Code availability

The custom code developed to train and evaluate the models is available at GitHub: https://github.com/MaastrichtU-CDS/hn_cnn.
