# Supplementary material for: Image based prognosis in head and neck cancer using convolutional neural networks: a case study in reproducibility and optimization
Source: Sci Rep. 2023 Oct 24;13:18176. doi: 10.1038/s41598-023-45486-5 (PMC10598263; doi:10.1038/s41598-023-45486-5)
Supplement: Supplementary file 2 — Supplementary Information 2. [file 41598_2023_45486_MOESM2_ESM.docx]

Image based prognosis in head and neck cancer using convolutional neural networks: a case study in reproducibility and optimization

Pedro Mateus^1^*, Leroy Volmer^1^, Leonard Wee^2^, Hugo J.W.L. Aerts^3,4,5^, Frank Hoebers^1^, Andre Dekker^1^, Inigo Bermejo^1^

^1^Department of Radiation Oncology (Maastro), GROW School for Oncology and Reproduction, Maastricht University Medical Centre+, Maastricht, The Netherlands

^2^Clinical Data Science, Maastricht University, Maastricht, The Netherlands

^3^Artificial Intelligence in Medicine (AIM) Program, Mass General Brigham, Harvard Medical School, Boston, MA, USA

^4^Department of Radiology and Nuclear Medicine, Maastricht University Medical Center, Maastricht, the Netherlands

^5^Departments of Radiation Oncology and Radiology, Brigham and Women's Hospital, Dana-Farber Cancer Institute, Harvard Medical School, Boston, MA, USA

Corresponding author email: pedro.mateus@maastro.nl

**Table 1 – Patient demographic and tumor characteristics across the different centers.**

|  | | Canadian Institutions | | | | Dutch institutions |
| --- | --- | --- | --- | --- | --- | --- |
|  |  | HGJ (n=91) | CHUS (n=100) | HMR (n=41) | CHUM (n=65) | Maastro (n=137) |
| Age (years) | Mean (SD) | 61.1 (11.0) | 64.0 (10.0) | 66.6 (9.47) | 63.5 (9.2) | 62.0 (8.8) |
| Sex | Female | 17 (18.7 %) | 28 (28.0 %) | 10 (24.4 %) | 16 (24.6 %) | 26 (19.0 %) |
|  | Male | 74 (81.3 %) | 72 (72.0 %) | 31 (75.6 %) | 49 (75.4 %) | 111 (81.0 %) |
| Follow-up (days) | Mean (SD) | 1612.3 (754.1) | 1386.0 (562.3) | 1177.5 (606.0) | 1208.5 (346.9) | 2236.3 (1328.7) |
| Tumor Site | Hypopharynx | 4 (4.4 %) | 1 (1.0 %) | 7 (17.1 %) | 1 (1.5 %) | 0 (0.0 %) |
|  | Larynx | 14 (15.4 %) | 21 (21.0 %) | 9 (22.0 %) | 0 (0.0 %) | 49 (35.8 %) |
|  | Nasopharynx | 14 (15.4 %) | 6 (6.0 %) | 6 (14.6 %) | 2 (3.1 %) | 0 (0.0 %) |
|  | Oropharynx | 55 (60.4 %) | 72 (72.0 %) | 19 (46.3 %) | 57 (87.7 %) | 88 (64.2 %) |
|  | Unknown | 4 (4.4%) | 0 (0.0 %) | 0 (0.0 %) | 5 (7.7 %) | 0 (0.0 %) |
| TNM Stage | Stage I | 1 (1.1 %) | 3 (3.0 %) | 0 (0.0 %) | 0 (0.0 %) | 24 (17.5 %) |
|  | Stage II | 5 (5.5 %) | 17 (17.0 %) | 3 (7.3 %) | 2 (3.1 %) | 11 (8.0 %) |
|  | Stage III | 28 (30.8 %) | 21 (21.0 %) | 5 (12.2 %) | 7 (10.8 %) | 23 (16.8 %) |
|  | Stage IV | 57 (62.6 %) | 60 (60.0 %) | 33 (80.5 %) | 54 (83.1 %) | 79 (57.7 %) |
|  | Unknown | 0 (0.0 %) | 0 (0.0 %) | 0 (0.0 %) | 2 (0%) | 0 (0.0 %) |

**Table 2 – Patient distribution for each outcome according to the follow-up time. Patients with a follow-up time below the defined event time were excluded.**

|  | | Training (n=191) | Validation (n=106) | Testing (n=137) |
| --- | --- | --- | --- | --- |
| Distant Metastasis  (2 years) | Follow-up time < 2 years | 8 (4.2 %) | 11 (10.4 %) | 28 (20.4 %) |
|  | Total | 183 (95.8 %) | 95 (89.6 %) | 109 (79.6 %) |
| Loco-regional Failure  (2 years) | Follow-up time < 2 years | 12 (6.3 %) | 9 (8.5 %) | 16 (11.7 %) |
|  | Total | 179 (93.7 %) | 97 (91.5 %) | 121 (88.3 %) |
| Overall Survival  (4 years) | Follow-up time < 4 years | 81 (42.4 %) | 52 (49.1 %) | 2 (1.5 %) |
|  | Total | 110 (57.6 %) | 54 (50.9 %) | 135 (98.5 %) |

**Table 3 – AUCs of the CNN models trained with clinical data for feature selection. Individual results consist of the mean AUC and range for the 5-fold cross validation.**

|  | Sex | Age | Location | Group stage | N-stage | T-stage | Volume | Area |
| --- | --- | --- | --- | --- | --- | --- | --- | --- |
| Imaging | 0.832 [0.76, 0.90] | 0.846 [0.74, 0.92] | 0.846 [0.75, 0.90] | 0.834 [0.73, 0.91] | **0.856 [0.72, 0.95]** | 0.818 [0.77, 0.87] | 0.840 [0.64, 0.96] | 0.786 [0.71, 0.87] |
| Imaging + N-stage | 0.856 [0.61, 0.97] | 0.848 [0.62, 0.97] | 0.852 [0.65, 0.96] | 0.846 [0.76, 0.92] | - | **0.864 [0.80, 0.92]** | 0.852 [0.73, 0.92] | 0.854 [0.74, 0.92] |
| Imaging + N-stage + Tstage | 0.836 [0.74, 0.95] | 0.860 [0.72, 0.95] | 0.850 [0.79, 0.95] | 0.844 [0.77, 0.96] | - | - | **0.868 [0.79, 0.94]** | 0.858 [0.78, 0.93] |
| Imaging + N-stage + T-stage + Volume | 0.846 [0.80, 0.90] | 0.816 [0.76, 0.90] | 0.808 [0.66, 0.89] | 0.850 [0.76, 0.94] | - | - | - | 0.800 [0.70, 0.89] |

**Table 4 – AUCs of the logistic regression models trained individually for each outcome.**

|  | | Cohort Split (CI 95%) | 5-fold CV |
| --- | --- | --- | --- |
|  |  |  | Mean (Range) |
| Distant Metastasis  (2 years) | Training | 0.82 [0.73, 0.89] | 0.81 (0.79-0.82) |
|  | Validation | 0.74 [0.58, 0.88] | 0.77 (0.71-0.89) |
|  | Testing | 0.86 [0.74, 0.95] | 0.85 (0.84-0.86) |
| Loco-regional Failure  (2 years) | Training | 0.64 [0.50, 0.75] | 0.65 (0.62-0.67) |
|  | Validation | 0.51 [0.33, 0.71] | 0.48 (0.35-0.61) |
|  | Testing | 0.49 [0.36, 0.62] | 0.51 (0.46-0.59) |
| Overall Survival  (4 years) | Training | 0.70 [0.59, 0.81] | 0.71 (0.68-0.74) |
|  | Validation | 0.66 [0.50, 0.81] | 0.67 (0.58-0.78) |
|  | Testing | 0.71 [0.62, 0.80] | 0.72 (0.71-0.73) |

**Table 5 - Comparative performance (AUCs) for the reproduced studies employing different windowing options.**

|  | Diamant et al.* - Cohort split mean (std) | | Lombardo et al. – 3-fold CV mean (range) | |
| --- | --- | --- | --- | --- |
|  | Level 0; Width 1000 | Level 125; Width 350 | Level 0; Width 1000 | Level 125; Width 350 |
| Training | 0.78 (0.09) | 0.87 (0.02) | 0.79 [0.70, 0.85] | 0.82 [0.78, 0.87] |
| Validation | 0.75 (0.02) | 0.85 (0.03) | 0.77 [0.72, 0.82] | 0.79 [0.73, 0.84] |
| Testing | 0.81 (0.06) | 0.85 (0.04) | 0.81** [0,72, 0,90] | 0.82** [0.73, 0.95] |
| *calculated for three CNN trainings by window option  **median (CI 83%) | | | | |
